# ADJUDICATION PILOT STUDY OF CAUSE OF DEATH IN THE LONG LIFE FAMILY STUDY

**DOI:** 10.1093/geroni/igz038.3372

**Published:** 2019-11-08

**Authors:** Habibatou Diallo, Joanne Murabito, Anne B Newman, Thomas T Perls, Diane Ives, Larry Honig

**Affiliations:** 1 Boston University Medical Campus, Boston, Massachusetts, United States; 2 Boston University, Boston, Massachusetts, United States; 3 University of Pittsburgh, Pittsburgh, Pennsylvania, United States; 4 Columbia University, New York, New York, United States

## Abstract

Background: Death certificate inaccuracy increases at older ages. The Long Life Family Study (LLFS) utilizes a physician adjudication committee to review the death certificate, medical records and a family narrative about cause of death. We report here the adjudication process and the prevalent underlying causes of death for a subsample of those who have died so far. Methods: We first describe the adjudication process. There were ~1,250 deaths in LLFS. We report underlying causes of death for a subset of proband generation subjects enrolled and evaluated by two LLFS study centers. Results: As of May 2019, we have adjudicated 190 deaths (98 male, 92 female) . Mean age 95 years (range 81-105 years). Top 5 causes of death for men: cancer (13%), coronary heart disease (CHD, 13%), dementia (13%), "other" (11%) and "unknown" (9%) and for women: dementia (21%), valvular heart disease (14%), coronary heart disease (12%), unknown (12%) and other (9%). Rate of death due to dementia was greater in women compared to men (CHI2 =7.33, p=0.006). Conclusions: In this pilot study, a significantly greater proportion of women died due to dementia compared to men. At least some portion of this difference may be due to the observation that women are known to survive chronic aging-related diseases more than men and thus have a greater opportunity to die from dementia at advanced ages. An additional cause to consider includes clinicians’ gender bias in ascribing diagnoses in the medical records that were relied upon as part of the adjudication process.

